# Associations between self-management strategies and clinical outcomes in depression: a cross-sectional survey

**DOI:** 10.3389/fpsyt.2026.1790462

**Published:** 2026-02-24

**Authors:** Teruo Tada, Hitoshi Sakurai, Taku Maruki, Masami Murao, Yasuyuki Matsumoto, Yumi Aoki, Hisateru Tachimori, Yayoi Imamura, Yu Matsumoto, Norifusa Sawada, Seiji Hongo, Sojiro Makino, Koji Tada, Takashi Tsuboi, Koichiro Watanabe

**Affiliations:** 1 Department of Neuropsychiatry, Kyorin University School of Medicine, Tokyo, Japan; 2 Psychiatric and Mental Health Nursing, Graduate School of Nursing Science, Saint Luke’s International University, Tokyo, Japan; 3 Department of Health Policy and Management, Keio University School of Medicine, Tokyo, Japan; 4 Kitanara Ekiue Hotto Clinic, Chiba, Japan; 5 Mental and Sleep Clinic of Narimasu, Tokyo, Japan; 6 Ichigaya Himorogi Clinic, Tokyo, Japan; 7 Makino Clinic, Tokyo, Japan; 8 Jimbocho Mental Health Clinic, Tokyo, Japan

**Keywords:** behavioral activation, personal recovery, psychoeducation, questionnaire survey, self-management

## Abstract

**Background:**

Self-management is essential for coping with depressive symptoms, yet little is known about its associations with depressive symptoms and personal recovery.

**Methods:**

We conducted a cross-sectional questionnaire survey of 183 outpatients with major depressive disorder in Japan between October 2024 and June 2025. Participants reported the use and perceived usefulness of 63 self-management strategies. Associations between the number of strategies and symptom severity and recovery were examined using Spearman’s rank correlation coefficients with the Japanese versions of the Quick Inventory of Depressive Symptomatology (QIDS-J) and the Questionnaire about the Process of Recovery (QPR-J).

**Results:**

Of the 183 respondents (mean age 43.6 ± 12.4 years; 54.1% female), the mean QIDS-J score was 9.55 ± 5.25 and 60.7% were in remission. Fourteen of the 63 strategies were used by at least half of the participants. The most frequently used strategies were “ensure enough rest to avoid exhaustion through overexertion” (88.0%), “engage in leisure activities (e.g. reading, watching TV, cooking, traveling, and driving)” (78.9%), and “eat a healthy diet” (65.0%). The strategies rated most useful included “discuss information about depression with a therapist” (86.7%), “meet with friends with whom I can be myself” (83.5%), and “avoid or minimize contact with people who tend to make me stressed or depressed” (83.3%). Participants reported using an average of 20.8 ± 13.5 strategies, of which 13.1 ± 9.16 were rated as useful. The number of strategies rated as useful was weakly negatively correlated with the QIDS-J scores (Spearman’s ρ = −0.207, p = 0.006) and moderately positively correlated with the QPR-J scores (Spearman’s ρ = 0.472, p < 0.001).

**Conclusion:**

The number of self-management strategies rated as useful showed a stronger correlation with personal recovery than with depressive symptom severity. The use of a greater number of self-management strategies may be associated with higher levels of personal recovery.

## Introduction

Self-management refers to the actions and skills through which individuals actively engage in coping with and regulating their chronic illnesses or symptoms in daily life (1). Such strategies are widely adopted by people living with chronic conditions, including diabetes (2), heart failure (3), emphysema (4), and asthma (5), and are known to contribute to healthier lifestyles and improved disease management. Importantly, self-management has been shown to enhance patients’ perceived control over their illness, a factor considered central to supporting personal recovery (6).

Self-management for coping with and managing symptoms is also essential for depression. The National Institute for Health and Care Excellence guidelines recommend self-management interventions, such as guided self-help, as an initial treatment option for non-severe depression (7). The Canadian Network for Mood and Anxiety Treatments (CANMAT) 2023 updated guidelines likewise emphasize the role of self-management in both the acute and maintenance phases of treatment for major depressive disorder (MDD) (8). Regarding the specific strategies employed by patients with depression, several investigations have been conducted. In one study, structured interviews with 20 patients in remission from MDD identified 50 distinct self-management strategies, whose utilization and perceived usefulness were subsequently evaluated in an additional 193 patients in remission (9, 10). Another study involving 25 patients with treatment-resistant depression identified 50 self-management strategies, of which 10 were regarded as particularly important (11). A review synthesizing 2214 self-help strategies from 93 sources identified 282 strategies considered useful for managing depressive symptoms (12). Among these, 48 strategies were endorsed by over 80% of 63 patients with depression and 34 mental health practitioners. The utilization and perceived usefulness of these strategies were further assessed in a large-scale online survey of 1,326 individuals with subthreshold depression (13). Across these studies, commonly endorsed strategies included engaging in physical activity, going out regularly, improving sleep hygiene and daily routines, ensuring adequate rest, and seeking support from trusted others.

However, most prior research has been conducted in Europe, North America, Australia, and New Zealand. Self-management strategies are likely to be shaped by cultural context and lifestyle, yet the practices of patients with depression in Asia have not been examined. In addition, variations in the use and perceived usefulness of self-management may depend on clinical symptoms and social functioning. Previous studies have generally focused on specific populations, such as patients in remission or those with treatment-resistant depression. As a result, little is known about how self-management is associated with clinical outcomes in broader patient populations.

The present study therefore aimed to identify the self-management strategies used by patients with depression in Japan and to evaluate their perceived usefulness in daily life. Additionally, the study sought to examine the associations between self-management and clinical outcomes by including patients at different phases of illness, from the acute phase to remission.

## Methods

### Overview

This cross-sectional, questionnaire-based survey was conducted between October 2024 and June 2025 among patients with MDD attending outpatient clinics at six institutions in Japan. The study was reviewed and approved by the School of Medicine Research Ethics Committee, Kyorin University, Tokyo, Japan. All participants received a full explanation of the study and provided written informed consent prior to participation.

### Participants

Eligible participants were outpatients aged 18 to 65 years who had been diagnosed with MDD according to the Diagnostic and Statistical Manual of Mental Disorders, Fifth Edition, Text Revision. Eligibility was not restricted to those experiencing a current depressive episode. Exclusion criteria included a diagnosis of bipolar disorder or dementia, the presence of psychotic symptoms, alcohol or substance use disorders, and severe physical illness.

### Questionnaire on self-management

Based on a review of previous studies on self-management strategies for mood disorders, we initially identified strategies that had demonstrated relatively high effectiveness and were considered feasible within the Japanese context. Strategies with substantial conceptual overlap were integrated through discussions among a multidisciplinary expert panel, which included physicians and nurses with clinical experience in mood disorders. Furthermore, strategies with limited applicability—such as those requiring current employment status or pet ownership—were excluded to enhance generalizability across diverse patient populations. Through this iterative consensus process involving multiple healthcare professionals, a final set of 63 strategies included in the questionnaire. Participants indicated whether they had used each strategy (“used” or “not used”). For strategies reported as “used,” participants rated their perceived usefulness on a four-point scale: not useful at all, slightly useful, moderately useful, or very useful. In addition, participants selected from among the following eight symptoms that they perceived as alleviated by self-management: depressed mood, loss of interest/anhedonia, anxiety, agitation, feelings of worthlessness, fatigue/loss of energy, diminished concentration, and suicidal ideation.

### Other measures

The questionnaire also included items on demographic characteristics, including age, sex, educational level, employment status, marital status, living arrangement, and religious affiliation. Participants completed two self-report scales: the Japanese versions of the Quick Inventory of Depressive Symptomatology (QIDS-J) (14) and the Questionnaire about the Process of Recovery (QPR-J) (15). The QPR-J consists of 22 items rated on a 5-point Likert scale, yielding a total score ranging from 22 to 110, with higher scores indicating greater personal recovery. Attending psychiatrists provided additional clinical information, including age at onset of the first depressive episode, presence of a current depressive episode, recurrence status, duration of depressive episodes, duration of remission, comorbid psychiatric disorders, and prescribed psychotropic medications. They also evaluated functioning using the Social and Occupational Functioning Assessment Scale (SOFAS). The SOFAS assesses overall social and occupational functioning independently of symptom severity, with scores ranging from 0 to 100, where higher scores reflect better functioning.

### Statistical analysis

The primary outcomes of the present study were the utilization rate and usefulness rate of each self-management strategy. The utilization rate was defined as the proportion of participants who reported using a given strategy. The usefulness rate was defined as the proportion of participants who rated the strategy as “moderately useful” or “very useful” among those who had used it. Spearman’s rank correlation coefficients were calculated to examine associations between scores on the QIDS-J, QPR-J, and SOFAS and the number of self-management strategies each participant reported as “used.” Similarly, correlations between these scores and the number of strategies each participant rated as “useful” (i.e. “moderately useful” or “very useful”) were examined. Following the correlation analyses, multivariable linear regression analyses were conducted for outcomes that showed significant correlations with the number of self-management strategies. The number of strategies was entered as an explanatory variable in regression models to examine its association with clinical outcomes after adjustment for potential confounders. Two hierarchical models were specified for each analysis. Model 1B was adjusted for age and sex, and Model 2B was further adjusted for comorbidity status (presence or absence of comorbid conditions) and educational attainment (university graduate vs. non-university graduate). In addition, covariates included in the multivariable models were determined based on the results of the correlation analyses. When the number of self-management strategies showed significant correlations with multiple clinical outcomes, other correlated outcome variables were included as covariates, as appropriate, to adjust for their interrelated effects. All analyses were performed by IBM SPSS Statistics, version 29.0 (IBM Corp., Armonk, NY, USA).

## Results

### Participant characteristics

A total of 190 patients were invited to participate, and valid responses were obtained from 183 individuals. Demographic and clinical characteristics of the respondents are presented in **Table 1**. The mean age was 43.6 ± 12.4 years, and 99 participants (54.1%) were female. Of the total sample, 111 (60.7%) were in remission and 72 (39.3%) were not. Recurrent depression was present in 103 participants (56.3%). The mean QIDS-J score was 9.55 ± 5.25.

**Table 1:** Demographic and clinical characteristics (n=183).

Characteristic	Mean ± SD or n (%)	Characteristic	Mean ± SD or n (%)
Age (years)	43.6 ± 12.4	Age at onset of first episode (years)	36.7 ± 11.8
Sex, female	99 (54.1%)	Remission	111 (60.7%)
Educational level		Duration of current episode (months)	36.3 ± 48.1
Junior high school	3 (1.6%)	Duration of remission (months)	22.3 ± 26.4
High school	35 (19.1%)	Recurrence	103 (56.3%)
Vocational school	31 (16.9%)	Medication	
University	110 (60.1%)	Antidepressants	158 (86.3%)
Employment status		Antipsychotic	55 (30.1%)
Regular employment	93 (50.8%)	Mood stabilizers	9 (4.9%)
Non-regular employment	36 (19.7%)	Benzodiazepines	81 (44.3%)
Supported employment	2 (1.1%)	Z-drugs	40 (21.9%)
Unemployed	29 (15.8%)	Other hypnotics	55 (30.1%)
Full-time homemaker	17 (9.3%)	Comorbidity	
Student	5 (2.7%)	Anxiety disorder	9 (4.9%)
Marital status		Panic disorder	8 (4.4%)
Married	78 (42.6%)	ASD	6 (3.3%)
Unmarried/divorced/widowed	104 (56.8%)	ADHD	8 (4.4%)
Living arrangement		Others	7 (3.8%)
Living with others	125 (68.3%)	QIDS-J score	9.55 ± 5.25
Living with alone	57 (31.1%)	SOFAS score	65.7 ± 16.8
Religious affiliation	7 (3.8%)	QPR-J score	70.7 ± 14.2

ADHD, Attention-Deficit/Hyperactivity Disorder; ASD, Autism Spectrum Disorder; QIDS-J, Quick Inventory of Depressive Symptomatology – Japanese version; QPR-J, Questionnaire about the Process of Recovery – Japanese version; SOFAS, Social and Occupational Functioning Assessment Scale.

### Utilization and usefulness of self-management strategies

The self-management strategies with the highest utilization and usefulness rates are shown in **Tables 2**, **3**. Among the 63 strategies, 14 were used by at least half of the participants. The most frequently used strategies were “ensure enough rest to avoid exhaustion through overexertion” (88.0%), “engage in leisure activities (e.g. reading, watching TV, cooking, traveling, and driving)” (78.9%), and “eat a healthy diet” (65.0%). In contrast, the strategies rated as most useful were “discuss information about depression with a therapist” (86.7%), “meet with friends with whom I can be myself” (83.5%), and “avoid or minimize contact with people who tend to make me stressed or depressed” (83.3%). Three strategies overlapped between the top 10 most frequently used and the top 10 most useful: “ensure enough rest to avoid exhaustion through overexertion,” “engage in moderate physical activity (e.g. cycling and walking),” and “maintain a regular day–night rhythm.” A complete list of utilization and usefulness rates for all strategies is provided in **Supplementary Tables 1**, **S2**.

**Table 2:** Self-management with high utilization.

Self-management	Utilization (%)	Usefulness (%)
Ensure enough rest to avoid exhaustion through overexertion	88.0	81.4
Engage in leisure activities (e.g. reading, watching TV, cooking, traveling, driving)	78.9	71.5
Eat a healthy diet	65.0	73.9
Engage in moderate physical activity (e.g. cycling, walking)	62.3	77.2
Ensure I am awakened every morning	59.0	63.9
Maintain a regular day–night rhythm	57.4	76.2
Have daily exposure to natural sunlight	55.7	74.5
Do something I usually enjoy, even if I do not feel like it	54.6	61.0
Avoid activities that tend to cause stress or low mood	54.1	73.7
Take a shower every day	52.5	63.5
Take the signs of my depression seriously	52.5	57.3

**Table 3:** Self-management with high perceived usefulness.

Self-management	Utilization (%)	Usefulness (%)
Discuss information about depression with a therapist	24.6	86.7
Meet with friends with whom I can be myself	46.4	83.5
Avoid or minimize contact with people who tend to make me stressed or depressed	36.1	83.3
Plan a holiday suited to my circumstances in consultation with my family or friends	31.7	82.8
Ensure enough rest to avoid exhaustion through overexertion	88.0	81.4
Talk over problems or feelings with someone supportive and caring	36.1	80.3
Avoid isolating myself	24.6	77.8
Practice good sleep hygiene	50.8	77.4
Engage in moderate physical activity (e.g. cycling, walking)	62.3	77.2
Maintain a regular day–night rhythm	57.4	76.2

### Symptoms addressed by self-management strategies

**Figure 1:**
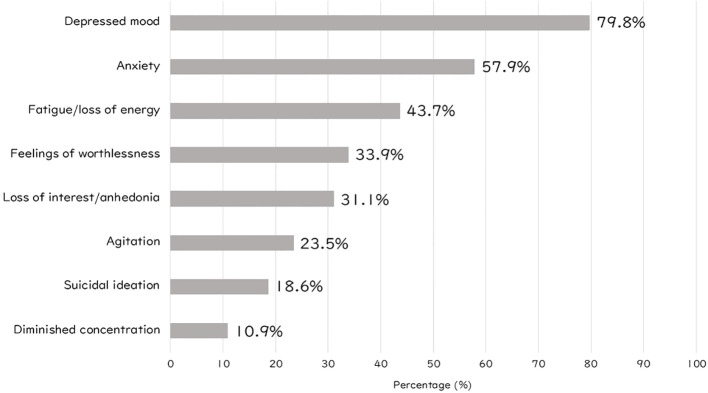
Percentage of self-management use for individual depressive symptoms.

**Figure 1** presents the percentages of eight psychiatric symptoms for which participants reported using self-management strategies. Within the study population, “depressed mood” was the most frequently targeted symptom (79.8%), followed by “anxiety” (57.9%).

### Correlations between the number of self-management strategies and clinical outcomes

Participants reported using an average of 20.8 ± 13.5 strategies, of which an average of 13.1 ± 9.16 were rated as “useful”. The number of strategies reported as “used” was not significantly correlated with the SOFAS or QIDS-J scores, but showed a weak positive correlation with the QPR-J scores (Spearman’s ρ = 0.332, p < 0.001). In contrast, the number of strategies rated as “useful” was not associated with the SOFAS scores, but demonstrated a weak negative correlation with the QIDS-J scores (Spearman’s ρ = −0.207, p = 0.006) and a moderate positive correlation with the QPR-J scores (Spearman’s ρ = 0.472, p < 0.001). In multivariable linear regression analyses (**Table 4**), the association between the number of self-management strategies rated as “useful” and QIDS-J scores observed in the correlation analysis was no longer statistically significant after adjustment for confounders in Model 2B. In contrast, the number of self-management strategies showed consistent associations with QPR-J scores. Both the number of strategies reported as “used” and the number rated as “useful” remained significantly associated with higher QPR-J scores after adjustment for age, sex, educational attainment, comorbidity status, and QIDS-J. These associations were robust across both adjusted models.

**Table 4:** Multivariable linear regression analyses for outcomes QIDS and QPR.

Independent variable	Outcome	Model 1B (95% CI)	p value	Model 2B (95% CI)	p value	Model 1B Adjusted R^2^	Model 2B Adjusted R^2^
The number of used strategies	QIDS	-0.007 (-0.063, 0.049)	0.819	0.036 (-0.011, 0.082)	0.130	0.07	0.42
The number of useful strategies	QIDS	-0.10 (-0.19, -0.021)	0.014	0.045 (-0.031, 0.12)	0.245	0.10	0.42
The number of used strategies	QPR	0.20 (0.048, 0.35)	0.010	0.21 (0.083, 0.33)	0.001	0.05	0.41
The number of useful strategies	QPR	0.69 (0.49, 0.90)	<0.001	0.53 (0.36, 0.70)	<0.001	0.23	0.49

Model 1 was adjusted for age and sex. Model 2 was further adjusted for educational level and comorbidity.

In Model 2 only, when QIDS was the outcome, QPR was additionally included as a covariate, and vice versa.

## Discussion

This study is the first cross-sectional survey to examine self-management strategies among patients with depression in Japan and to evaluate their perceived usefulness in daily life. Strategies such as “ensure enough rest to avoid exhaustion through overexertion”, “engage in moderate physical activity (e.g. cycling and walking)”, and “maintain a regular day–night rhythm” demonstrated both high utilization and usefulness rates. These findings underscore the importance of promoting adequate rest, physical activity, and lifestyle regularity as part of routine clinical care and psychoeducation for depression. Moreover, the number of strategies was positively associated with personal recovery. This finding suggests that having a broader repertoire of self-management strategies may be linked to with more favorable clinical outcomes in depression.

In this study, both lifestyle strategies, including rest, diet, exercise, and regulation of daily rhythms, and behavioral activation strategies, including engaging in leisure and enjoyable activities, were frequently used. Specifically, participants most often reported “ensure enough rest to avoid exhaustion through overexertion” (88.0%), “eat a healthy diet” (65.0%), “engage in moderate physical activity (e.g. cycling and walking)” (62.3%), “ensure I am awakened every morning” (59.0%), “maintain a regular day-night rhythm” (57.4%), “have daily exposure to natural sunlight” (55.7%), “engage in leisure activities (e.g. reading, watching TV, cooking, traveling, and driving)” (78.9%), and “do something I usually enjoy, even if I do not feel like it” (54.6%). The CANMAT 2023 updated guidelines recommend skills such as problem solving, lifestyle modification, and behavioral activation as important components of illness management. Similarly, the clinical guidelines for MDD by the Japanese Society of Mood Disorders emphasizes lifestyle modifications, especially sleep hygiene (16). These recommendations may help explain the high utilization rates of lifestyle and behavioral activation strategies observed in the present study. Furthermore, previous studies in other countries have also reported high utilization of these strategies, indicating that they are widely adopted by individuals with depression regardless of cultural background or treatment settings (10–13).

Regarding perceived usefulness, self-management strategies related to social connections and supportive relationships were among those rated most highly. For example, “discuss information about depression with a therapist” (86.7%), “meet with friends with whom I can be myself” (83.5%), “avoid or minimize contact with people who tend to make me stressed or depressed” (83.3%), “talk over problems or feelings with someone supportive and caring” (80.3%), and “avoid isolating myself” (77.8%) were rated “useful” by participants. A systematic review of longitudinal studies, including 23 reports sampling individuals with depression, found that loneliness and low social support were associated with poorer clinical outcomes (17). These findings highlight the importance of strategies that focus on seeking support from others and preventing loneliness. However, in our study, the utilization rates of such strategies were relatively low, suggesting that they are not widely adopted by patients with depression. This pattern may reflect tendencies toward social avoidance or self-stigma (18, 19), which are commonly observed in this population. Although these strategies appear useful, they are not sufficiently recognized by patients, and therefore greater efforts are needed to increase awareness and promote their dissemination. At the same time, initiatives that broaden access to supportive interpersonal environments in which individuals feel safe and accepted, together with guidance on building and maintaining relationships, may play an important role in facilitating the use of these strategies.

The number of self-management strategies reported as “used” was weakly positively correlated with the QPR-J scores. Furthermore, the number of self-management strategies rated as “useful” showed a significant moderate positive association with the QPR-J scores. These associations remained statistically significant in multivariable linear regression analyses after adjustment for potential confounders, indicating the robustness of the observed relationships. Although differences in correlation strength alone do not allow firm conclusions regarding differential clinical impact, the present findings consistently indicate that employing more self-management strategies perceived as effective, rather than simply employing many strategies, is associated with higher personal recovery. In recent studies, it has been emphasized that treatment of depression should move beyond the exclusive pursuit of clinical recovery. While symptom reduction remains a central treatment goal, it does not necessarily equate to recovery from the patient’s perspective, and growing evidence highlights the importance of personal recovery, which encompasses subjective well-being, meaning in life, and a sense of agency (20, 21). In this context, the present results indicate that self-management is relevant not only for alleviating depressive symptoms but also for promoting higher levels of personal recovery. The practice of self-management strategies might lead to changes in self-recognition and agency, thereby promoting personal recovery. Among individuals with depression who have achieved clinical recovery but remain dissatisfied with their current state, expanding the repertoire of self-management strategies can help facilitate a more meaningful and patient-centered form of recovery.

It is noteworthy that 79.8% of participants reported self-management as effective for depressed mood and 57.9% for anxiety, both of which are commonly experienced symptoms of depression. In contrast, lower effectiveness rates were observed for diminished concentration (10.9%), suicidal ideation (18.6%), and agitation (23.5%). While self-management may be well-suited for common affective and anxiety-related symptoms, it may have limited utility in addressing cognitive impairments, acute risk states, or severe agitation, which require professional intervention.

The present study has several limitations. First, all data were based on self-report, limiting the findings to subjective evaluations and potentially introducing recall bias in participants’ reports of self-management strategy use. Furthermore, self-reported date may be influenced by social desirability bias, potentially resulting in the overestimation of strategies perceived as beneficial and the underreporting of those considered less socially acceptable. Second, because strategies were selected based on previous international studies, self-management strategies specific to the Japanese cultural context might not have been captured. Future studies should incorporate qualitative approaches to identify culturally specific strategies. Third, our sample did not include younger or older populations. Given that self-management needs and capacities differ across the lifespan, inclusion of broader age groups is necessary to improve generalizability. Finally, the cross-sectional design of this study does not allow for causal interpretation of the relationships between self-management and clinical outcomes. In particular, the observed associations could reflect reverse causation, whereby individuals with less severe symptoms or higher levels of personal recovery are more likely to engage in self-management strategies and to experience greater perceived benefits. Longitudinal and interventional studies are required to examine the directionality of these associations.

In conclusion, the present study identified the self-management strategies employed by individuals with depression in Japan. It revealed that even strategies perceived as highly useful are not necessarily widely practiced, highlighting the importance of disseminating information about such strategies to patients. Moreover, the findings indicate that broadening the repertoire of self-management strategies is associated with higher levels of personal recovery. Future research should further explore how self-management strategies can be effectively integrated into routine clinical practice and psychoeducation programs.

## Data Availability

The raw data supporting the conclusions of this article will be made available by the authors, without undue reservation.
